# Metagenomic analysis reveals distinct patterns of gut microbiota features with diversified functions in *C. difficile* infection (CDI), asymptomatic carriage and non-CDI diarrhea

**DOI:** 10.1080/19490976.2025.2505269

**Published:** 2025-05-14

**Authors:** Lamei Wang, Xinhua Chen, Nira R. Pollock, Javier A. Villafuerte Gálvez, Carolyn D. Alonso, Dangdang Wang, Kaitlyn Daugherty, Hua Xu, Junhu Yao, Yulin Chen, Ciaran P. Kelly, Yangchun Cao

**Affiliations:** a College of Animal Science and Technology, Northwest A&F University, Yangling, Shaanxi, China; b Division of Gastroenterology, Department of Medicine, Beth Israel Deaconess Medical Center, Harvard Medical School, Boston, MA, USA; c Division of Infectious Disease, Department of Medicine, Beth Israel Deaconess Medical Center, Harvard Medical School, Boston, MA, USA; d Department of Laboratory Medicine, Boston Children’s Hospital, Harvard Medical School, Boston, MA, USA

**Keywords:** CDI, metabolomics, gut microbiota, module, antibiotic resistance

## Abstract

*Clostridioides difficile* infection (CDI) has been recognized as a leading cause of healthcare-associated infections and a considerable threat to public health globally. Increasing evidence suggests that the gut microbiota plays a key role in the pathogenesis of CDI. The taxonomic composition and functional capacity of the gut microbiota associated with CDI have not been studied systematically. Here, we performed a comprehensive shotgun metagenomic sequencing in a well-characterized human cohort to reveal distinct patterns of gut microbiota and potential functional features associated with CDI. Fecal samples were collected from 104 inpatients, including : (1) patients with clinically significant diarrhea and positive nucleic acid amplification testing (NAAT) and received CDI treatment (CDI, *n* = 47); (2) patients with positive stool NAAT but without diarrhea (Carrier, *n* = 17); (3) patients with negative stool NAAT but with diarrhea (Diarrhea, *n* = 14); and (4) patients with negative stool NAAT and without diarrhea (Control, *n* = 26). Downstream statistical analyses (including alpha and beta diversity analysis, differential abundance analysis, correlation network analysis, and potential functional analysis) were then performed. The gut microbiota in the Control group showed higher Chao1 index (*p* < 0.05), while Shannon index at KEGG module level was higher in CDI than in Carrier and Control (*p* < 0.05). Beta diversity for species composition differed significantly between CDI vs Carrier/Control cohorts (*p* < 0.05). Microbial Linear discriminant analysis Effect Size and ANCOM analysis both identified 8 species (*unclassified_f_Enterobacteriaceae, Veillonella_parvula*, *unclassified_g_Klebsiella* and etc.) were enriched in CDI, *Enterobacter_aerogenes* was enriched in Diarrhea, *Collinsella_aerofaciens*, *Collinsella_sp_4_8_47FAA*, *Collinsella_tanakaei* and *Collinsella_sp_CAG_166* were enriched in Control (LDA >3.0, adjusted *p* < 0.05). Correlation network complexity was higher in CDI with more negative correlations than in other three cohorts. Modules involved in iron complex transport system (M00240) was enriched in CDI, ABC-2 type transport system (M00254), aminoacyl-tRNA biosynthesis (M00359), histidine biosynthesis (M00026) and inosine monophosphate biosynthesis (M00048) were enriched in Carrier, ribosome (M00178 and M00179) was enriched in Diarrhea, fluoroquinolone resistance (M00729) and aminoacyl-tRNA biosynthesis (M00360) were enriched in Control (LDA > 2.5, adjusted *p* < 0.05). Resistance functions of acriflavine and glycylcycline were enriched in CDI, while resistance function of bacitracin was enriched in Carrier (LDA > 3.0, adjusted *p* < 0.05), and the contributions of phylum and species to resistance functions differed among the four groups. Our results reveal alterations of gut microbiota composition and potential functions among four groups of differential colonization/infection status of *Clostridioides difficile*. These findings support the potential roles of gut microbiota and their potential functions in the pathogenesis of CDI.

## 1. Introduction

*Clostridioides difficile* (*C. difficile*) is a gram-positive, spore-forming, toxin-producing anaerobic bacterium.^1^ It is a leading nosocomial enteric pathogen that causes significant human morbidity and mortality, resulting in half a million infections and roughly 30,000 deaths per year and costing over $6.3 billion in healthcare expenses with nearly 2.4 million days of inpatient stay attributable to CDI annually in the United States.^2,3^ Outcomes of CDI hospitalizations improved over the studied decade, but CDI still places significant mortality and economic burden on the US health care system.^4^ A major risk factor in the pathogenesis of *C. difficile* infection (CDI) is the use of antibiotics,^5^ as these drugs lead to significant and long lasting shifts in the gut microbiota and metabolome, resulting in loss of colonization resistance against *C. difficile*.^6–8^ Although the exact mechanisms of colonization resistance against *C. difficile* remain unclear, there is increasing evidence that microbiota play an important role.

Understanding how alterations to the gut microbiota contribute to CDI susceptibility is expected to identify novel biomarkers that may predict treatment outcome and improve diagnostics.^9^ Asymptomatic colonization is estimated to be 2% in the general adult population and up to 26% in the health care-exposed individuals.^10^ The currently preferred diagnostic platforms, nucleic acid amplification of the *C. difficile* toxin-encoding genes, toxin immunoassay or glutamate dehydrogenase assay, cannot distinguish between colonization and disease.^11^ There is currently no clinical diagnostic available that quickly and reliably identifies patients at risk for CDI.

The human gut microbiota is an important microecosystem living in symbiosis with the human body. Currently, there are two main strategies for the analysis of the gut microbiome using next generation sequencing, shotgun metagenomics, and 16S rRNA gene sequencing. The metagenomics approach, in which all the DNA fragments in a sample are sequenced rather than only 16S rRNA amplicons, results in greater in-depth coverage and more informative sequencing datasets. Analyses of these datasets will help to elucidate the composition of microbial communities and are valuable resources for identifying potential functional features, such as carbohydrate active enzymes and antibiotics resistance genes present in gut communities. To the best of our knowledge, only very limited metagenomic analyses of the functional aspects of the gut microbiota in CDI have been reported.^7,12–15^ This scarcity is likely due to challenges in accessing well-documented CDI cohorts, the high cost of metagenomic sequencing, and the significant bioinformatic resources required for accurate functional profiling.

We hypothesize that gut microbial compositions and potential functions can serve for CDI diagnosis purposes. To test this hypothesis, we performed gut microbial compositions data analysis of 104 hospitalized individuals that consist of patients with CDI (*n* = 47), antibiotic-exposed Carrier (*n* = 17), non *C. difficile* Diarrhea (*n* = 14) and antibiotic-exposed asymptomatic noncarriers (*n* = 26). In this study, we aimed to compare the gut microbial compositions and potential functions using a comprehensive deep shotgun metagenomics sequencing approach among four well-characterized human clinical cohorts. The results of this study provide a deeper exploration of the gut microbiomes and potential functions of four cohorts. The data provide potential avenues for developing novel diagnostic tools and therapeutic strategies to better manage CDI and its associated complications, as well as in the underlying pathogenic mechanisms of CDI.

## 2. Materials and methods

### 2.1. Study participants

The background and design of this cohort has been detailed in our previous studies.^11,16,17^ A total of 412 patients were initially considered eligible for enrollment based on pre-screening. The majority were excluded because they did not meet all inclusion/exclusion criteria (details see supplementary methods). Finally, 104 samples met all inclusion/exclusion criteria and had sufficient volume for all study testing. All 104 participants individuals were adults (age >18 years old). CDI patients (*n* = 47) were inpatients with positive clinical stool NAAT result, new-onset diarrhea, and a decision to treat for CDI. The diagnostic clinical stool sample was captured as a discarded sample; a discarded serum sample collected within one day of that stool sample was also captured. Patients were excluded if the diagnostic stool specimen was more than 72 hours old, if they had received CDI treatment for more than 24 hours prior to stool collection, or if they had a colostomy. Carrier (*n* = 17) were admitted for at least 72 hours, had received at least one dose of non-CDI-directed antibiotic within the past 7 days, and did not have diarrhea in the 48 hours prior to stool sample collection, but had positive NAAT results on stool testing and were not treated for CDI. Patients with two or more loose stools within a 24-hour period were excluded; patients with one loose stool were included only if providers had recently administered a laxative. Patients were excluded if they had a colostomy; received oral or intravenous metronidazole, oral vancomycin, oral rifaximin, and/or oral fidaxomicin for more than 24 hours within the prior 7 days; had been diagnosed with CDI in the past 6 months; or had tested negative for *C. difficile* within the past 7 days. Stool samples were collected prospectively under verbal informed consent. A discarded serum sample from within one day of the stool sample was also captured. Diarrhea cohort (*n* = 14) had diarrhea (confirmed using the same definition used for the CDI cohort) but had tested NAAT negative on clinical *C. difficile* testing; stool samples were captured prior to discarding. Control groups (*n* = 26) included individuals without diarrhea who had screened as eligible for the Asymptomatic Carrier group but were NAAT negative on stool testing. Fecal samples were freshly collected from individuals and transported to the laboratory on ice. All samples were refrigerated immediately and stored at −80°C until use.

### 2.2. Shotgun metagenomic sequencing

DNA of fecal samples (200 mg) were extracted using Mag-Bind® Universal Metagenomics Kit (Product# M5633–01, Omega Biotek) and DNeasy PowerSoil Kit (Catalog# 12888–100, Qiagen) according to manufacturer’s instructions. Sequencing was then performed on the Illumina HiSeq X Ten platform, and 150 bp paired-end library was constructed for each sample. The quality of the library was examined by Agilent 2100 bioanalyzer. To guarantee data quality, low-quality raw reads were trimmed (fastp, V0.20.0). The sequence quality filtering process involved several steps and started by removing reads that contained 10% or more ambiguous bases. The adapter sequences were removed (15 bases or longer sequence aligned to the adapter sequence). The reads of less than 50 bp in length and an average quality score of less than 20 were removed. Furthermore, a filtering step is added to remove the sequence of the human genome so as to reduce the interference of the host sequence on the subsequent analysis using SOAP2.^18^ The fragments longer than 500 bp in all Scaftigs were used for further analysis. The high-quality reads of each sample were assembled by the SOAPdenovo software (V2.04), and MetaGeneMark (V2.10, http://topaz.gatech.edu/GeneMark/) was used to predict open reading frames (ORFs) from the Scaftigs assembled from each sample as well as the Scaftigs from the mixed assembly.^19^ CD-HIT software (V4.5.8, http://www.bioinformatics.org/cd-hit.) was used to reduce sequence redundancy and the unique initial gene catalog was obtained.^20^ Functional annotation of metagenomes was conducted using DIAMOND software (V2.0.13, http://www.diamondsearch.org/index.php.) to blast unigenes to the NCBI non-redundant protein database, Kyoto Encyclopedia of Genes and Genomes (KEGG) database, the antibiotic resistance genes database (ARDB, http://ardb.cbcb.umd.edu/.) and the pathogen-host interactions database (http://www.phi-base.org) with the parameter setting of blastp (e-value cutoff of 1e-5). The output files of nr BLAST were analyzed using MEGAN (V4.6, http://ab.inf.uni-tuebingen.de/software/megan4/).^21^ Details of fecal DNA extraction, shotgun metagenomic sequencing, quality control, read quality filtering, de novo assembly, gene prediction, taxonomic profiling, data processing and bioinformatics analysis are available online as supplementary methods.

### 2.3. Statistical analysis

Low-frequency and less abundant microbial features (species and potential functional modules) were discarded (online supplementary methods). Permutational multivariate analysis of variance (PERMANOVA) was performed with the default 999 permutations based on the Adonis (R package V3.3.1). Note that in the PERMANOVA tests, we only included subjects with known information of cohorts, sex, age, race, and ethnicity. Alpha diversity (Chao1 and Shannon index) was calculated to estimate microbial, Kyoto Encyclopedia of Genes and Genomes (KEGG) module or antibiotic resistance gene (ARG) diversity. Principal coordinates analysis (PCoA) with Bray-Curtis dissimilarity was used to examine the separation between samples at species, module and antibiotic resistance levels. Cluster analysis was performed to reveal the distinct bacterial signature among four cohorts using the heatmap package in R based on the differentially abundant species. Differences in relative abundance of the microbial features, module and antibiotic resistance were determined by linear discriminant analysis (LDA) effect size (LEfSe), with adjusted *p* < 0.05 and LDA > 3 (species and antibiotic resistance levels) or LDA > 2.5 (module level) considered significant level.^22^ ANCOM (analysis of composition of microbiomes) was conducted after removing spurious observations using default parameters with a Benjamini-Hochberg correction significance threshold of 0.05 (QIIME2 v2022.2).^23^ Microbial correlation network was constructed using SparCC (V1.1.0).^24^ Correlated species pairs were selected if the absolute value of sparse correlation |r| > 0.3 and adjusted *p* < 0.05. LEfSe, ANCOM and SparCC with the false discovery rate (Benjamini-Hochberg FDR) method was used to adjust the *p* values for multiple test correction. R software were used for data management and statistical analyses.

## 3. Results

### 3.1. Study population

In the present study, we enrolled 104 fecal samples from inpatients with CDI (*n* = 47), Carrier (*n* = 17), Diarrhea (*n* = 14) and Control (*n* = 26) individuals. The statistical analysis indicated that there were no significant differences in clinical characteristics (sex, age, race, and ethnicity) among the four cohorts (*p* > 0.05, Supplementary Table S1). Additionally, there were no significant differences between the elderly (age >65) and non-elderly (age <65) populations within the cohorts (*p* > 0.05, Supplementary Table S1). These statistical analyses suggested that the clinical characteristics of the participants were demographically comparable. PERMANOVA showed that cohorts and clinical characteristics of the participants had no significant effect on the fecal microbiomes’ composition (adjust *p* > 0.05, Supplementary Table S2). These findings suggest that the microbiome variations observed were independent of cohorts and clinical characteristics of the participants. In this study, approximately a total of 1042 billion bp raw data were generated with an average of 10.40 ± 1.34 (mean ± SD) billion bp raw data for each sample. After quality control, a total of 6.93 billion 150-bp paired-end high-quality reads free of adaptor and human DNA contaminants were generated, with an average of 66.66 ± 16.85 (mean ± SD) million reads for each sample (Supplementary Table S3).

### 3.2. Alpha and beta diversity

**Figure 1.**
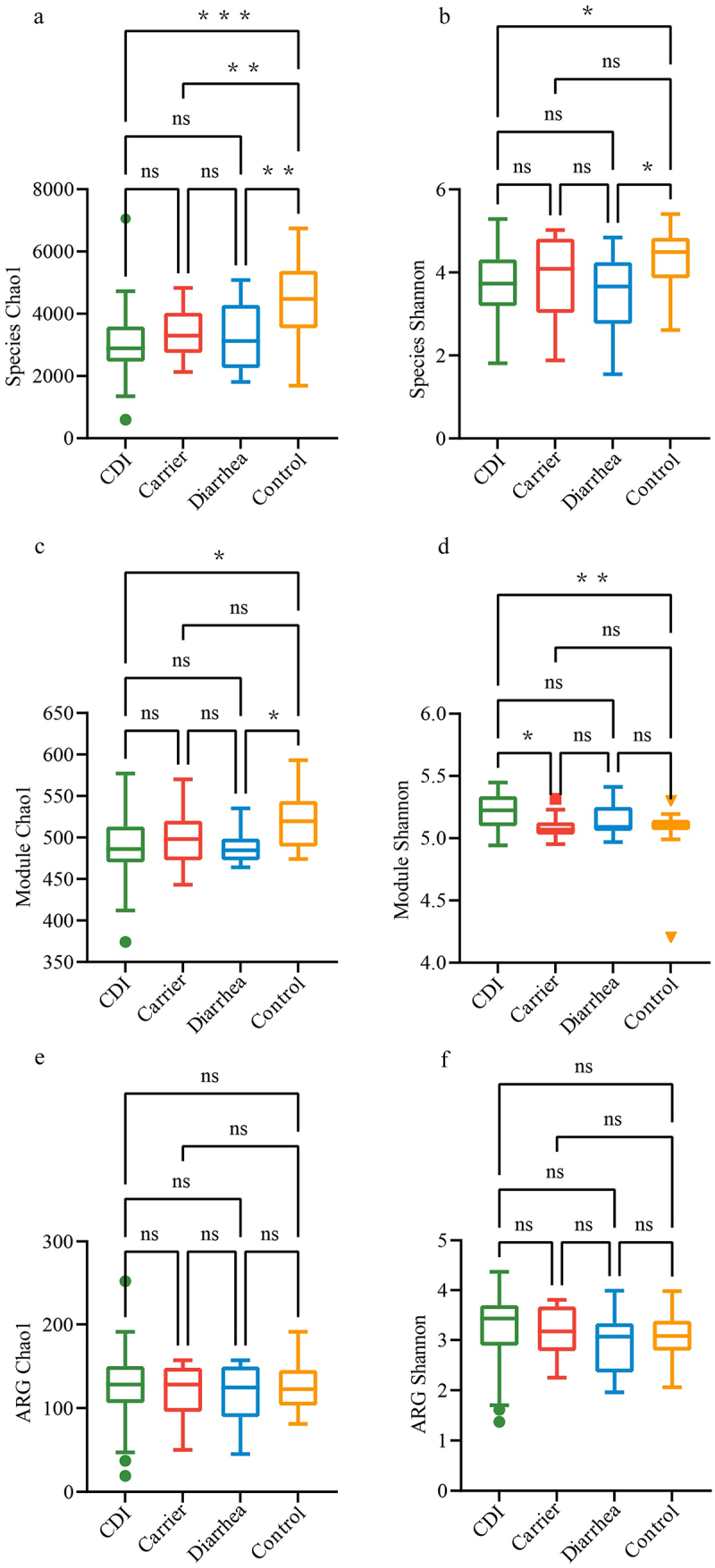
Alpha diversities of the fecal microbiota in CDI, carrier, diarrhea and control participants. Chao1 indices reflect the abundance species, module and antibiotic resistance gene (ARG) in samples, and Shannon indices reflect the diversity of species, module and antibiotic resistance gene in samples. Alpha diversity at species level, Chao1 index (a) and Shannon index (b). Alpha diversity at module level, Chao1 index (c) and Shannon index (d). Alpha diversity at antibiotic resistance gene level, Chao1 index (e) and Shannon index (f). Abbreviations: ARG, antibiotic resistance gene. ns: *p* > 0.05, *p < 0.05, ***p < 0.001.

**Figure 2.**
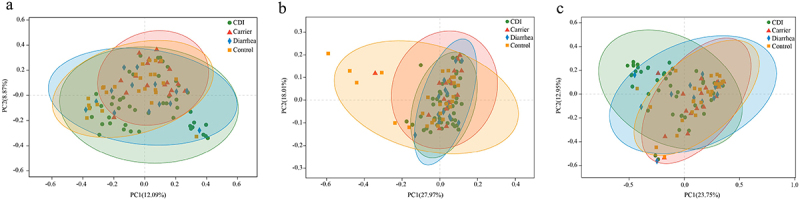
Beta diversities of the fecal microbiota in CDI, carrier, diarrhea and control participants. Principal coordinates analysis (PCoA) with Bray-Curtis dissimilarity was performed to assess the community structure at the species level (a), module level (b) or antibiotic resistance gene (ARG) level (c) in four groups. The ordinate and abscissa represent the two main coordinate axes, and the percentage values of the coordinate axes represent interpretations of the differences in sample composition. The closer the two sample points are, the more similar their bacteria composition. The ellipses represent the 95% of the samples belonging to each group. Dissimilarity was analyzed using adonis statistical tests with 999 permutations based on Bray-Curtis dissimilarity at the species level {CDI vs carrier (R^2^ = 0.125, *p* = 0.037), CDI vs diarrhea (R^2^ = 0.142, *p* = 0.037) and CDI vs control (R^2^ = 0.089, *p* = 0.030)} at the ARG level CDI vs control (R^2^ = 0.136, *p* = 0.003) while no significant differences were found among four cohorts at the modules level (*p* > 0.05). Abbreviations: ARG, antibiotic resistance gene; PCoA, principal coordinates analysis.

Based on the species profile, we calculated the within-sample (alpha) diversity to estimate gut microbiota richness and diversity based on the Chao1 index and Shannon index. The Chao1 index was higher for Control patients than CDI, Carrier and Diarrhea cohorts at species level (*p* < 0.05, Wilcoxon rank sum test, Figure 1(a)), and the Shannon index was higher for Control patients than CDI and Diarrhea cohorts at species level (*p* < 0.05, Wilcoxon rank sum test, Figure 1(b)). Chao1 index was higher for Control patients than CDI and Diarrhea cohorts at module level (*p* < 0.05, Wilcoxon rank sum test, Figure 1(c)). While Shannon index at module level was higher in CDI than in Carrier and Control (*p* < 0.05, Wilcoxon rank sum test, Figure 1(d)). Chao1 and Shannon index at ARG level showed no significant differences among four cohorts (*p* > 0.05, Wilcoxon rank sum test, Figure 1(e,f)). We further examined the between-sample (beta diversity) variability in fecal samples by performing PERMANOVA with Bray-Curtis distance. Gut microbial community structure at the species level differed significantly between CDI vs Carrier (R^2^ = 0.125, *p* = 0.037), CDI vs Diarrhea (R^2^ = 0.142, *p* = 0.037) and CDI vs Control (R^2^ = 0.089, *p* = 0.030, Figure 2(a)). However, no significant differences were found among four cohorts at the modules level (*p* > 0.05, Figure 2(b)). Beta diversity at antibiotic resistance level showed no significant differences (*p* > 0.05, Figure 2(c)), except CDI vs Control (R^2^ = 0.136, *p* = 0.003) which showed a significant difference.

### 3.3. CDI patients exhibit a distinct fecal microbiota

**Figure 3.**
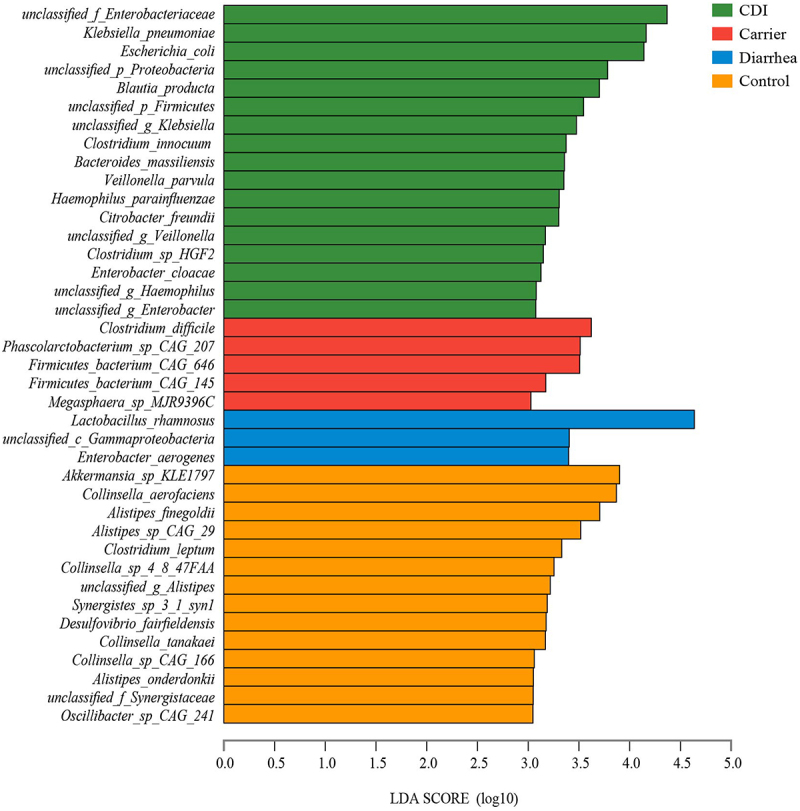
Different trends of species from the fecal microbiomes of CDI, carrier, diarrhea and control participants. Linear discriminant analysis (LDA) effect size (LEfSe) is a tool to identify biomarkers from high dimensional data of two or more groups using. This tool clarifies about statistical significance and biological correlation and can identify statistically different biomarkers between groups. Identified biomarkers ranked by effect size, and only species meeting an LDA significant threshold > 3.0 and adjusted *p* < 0.05 were shown. Abbreviations: LDA, linear discriminant analysis; LEfSe, linear discriminant analysis effect size.

To further explore features of the gut microbial community in four cohorts, we compared the relative abundances of the gut microbiota at the phylum and species levels. The main phyla in the fecal of four cohorts were Firmicutes, followed by Bacteroidetes and Proteobacteria (Supplementary Figure S1). And the main species were *unclassified_g_Bacteroides*, followed by *Akkermansia_sp._CAG:344*, *unclassified_f_Enterobacteriaceae*, *Escherichia_coli*, *Lactobacillus_rhamnosus* and *Enterococcus_faecium* (Supplementary Figure S2). To identify differentially abundant microbes between cohorts, we performed two analytical methods up to the species level: the LEfSe method and the ANCOM. The matching results between analytical methods increases the credibility of biological interpretation. Using the LEfSe method, we observed 39 species differed significantly among four cohorts: 17 species were enriched in CDI, including *unclassified_f_Enterobacteriaceae*, *Klebsiella_pneumoniae*, *Escherichia_coli*, *unclassified_p_Proteobacteria*, *Blautia_producta*, *unclassified_p_Firmicutes*, *unclassified_g_Klebsiella*, *Clostridium_innocuum*, *Bacteroides_massiliensis*, *Veillonella_parvula* etc.; five species were enriched in Carrier, including *C. difficile*, *Phascolarctobacterium_sp_CAG_207*, *Firmicutes_bacterium_CAG_646*, *Firmicutes_bacterium_CAG_145* and *Megasphaera_sp_MJR9396C*; three species were enriched in Diarrhea, including *Lactobacillus_rhamnosus*, *unclassified_c_Gammaproteobacteria* and *Enterobacter_aerogenes*; while 14 species were enriched in Controls, including *Akkermansia_sp_KLE1797*, *Collinsella_aerofaciens*, *Alistipes_finegoldii*, *Alistipes_sp_CAG_29*, *Clostridium_leptum*, *Collinsella_sp_4_8_47FAA* etc. (LDA >3, adjusted *p* < 0.05, Figure 3 and Supplementary Figure S3). To complement the LEfSe analysis, and to strengthen the results of the differential abundances, ANCOM analysis was performed. Confirming the LEfSe findings, ANCOM analysis also identified *Haemophilus_parainfluenzae*, *unclassified_g_Veillonella, unclassified_g_Enterobacter*, *Enterobacter_cloacae*, *Veillonella_parvula*, *Citrobacter_freundii*, *unclassified_g_Klebsiella* and *unclassified_f_Enterobacteriaceae* were enriched in CDI, *Enterobacter_aerogenes* was enriched in Diarrhea, *Collinsella_aerofaciens*, *Collinsella_sp_4_8_47FAA*, *Collinsella_tanakaei* and *Collinsella_sp_CAG_166* were enriched in Control (Supplementary Figure S4). Further classification at the species level, a hierarchical heat map of the relative abundance of top-50 most abundant species (Supplementary Figure S5) indicated that microbial communities of those four groups were unique.

### 3.4. Classification with random forests model

To illustrate the diagnostic power of fecal microbiota, we constructed a random forest classifier to distinguish four cohorts. The classification performance was evaluated by the area under the curve (AUC) of the receiver operating characteristic. we found that *Novosphingobium_fuchskuhlense* (AUC = 0.978, 95% CI [0.945, 1.000], CDI vs Carrier; AUC = 0.879, 95% CI [0.739, 1.000], Carrier vs Diarrhea), *unclassified_f_Peptostreptococcaceae* (AUC = 0.787, 95% CI [0.650, 0.922], CDI vs Diarrhea), *Flavobacterium_sp._KMS* (AUC = 0.893, 95% CI [0.810, 0.976], CDI vs Control), *Pseudomonas_putida* (AUC = 0.803, 95% CI [0.672, 0.933], Carrier vs Control) and *Citrobacter_sp._BIDMC107* (AUC = 0.879, 95% CI [0.955, 1.000], Diarrhea vs Control) were the most important microbial features (Supplementary Figure S6), respectively. Notably, combining microbial features sets reached a superior classification with AUC 1.000 (95% CI [1.000, 1.000], CDI vs Carrier), 0.913 (95% CI [0.843, 1.000], CDI vs Diarrhea), 0.981 (95% CI [0.957, 1.000], CDI vs Control), 1.000 (95% CI [1.000, 1.000], Carrier vs Diarrhea), 0.915 (95% CI [0.843, 1.000], Carrier vs Control) and 0.921 (95% CI [0.843, 1.000], Diarrhea vs Control), respectively (Supplementary Figure S6). The importance of each feature was quantified by the Mean Decrease in Accuracy (MDA) of the classifier due to the exclusion (or permutation) of this feature (Supplementary Figure S7). These results suggested that the random forest classifier based on combined microbial features can achieve a powerful diagnostic performance in differentiating four group.

### 3.5. CDI alters microbial potential functional characteristics

**Figure 4.**
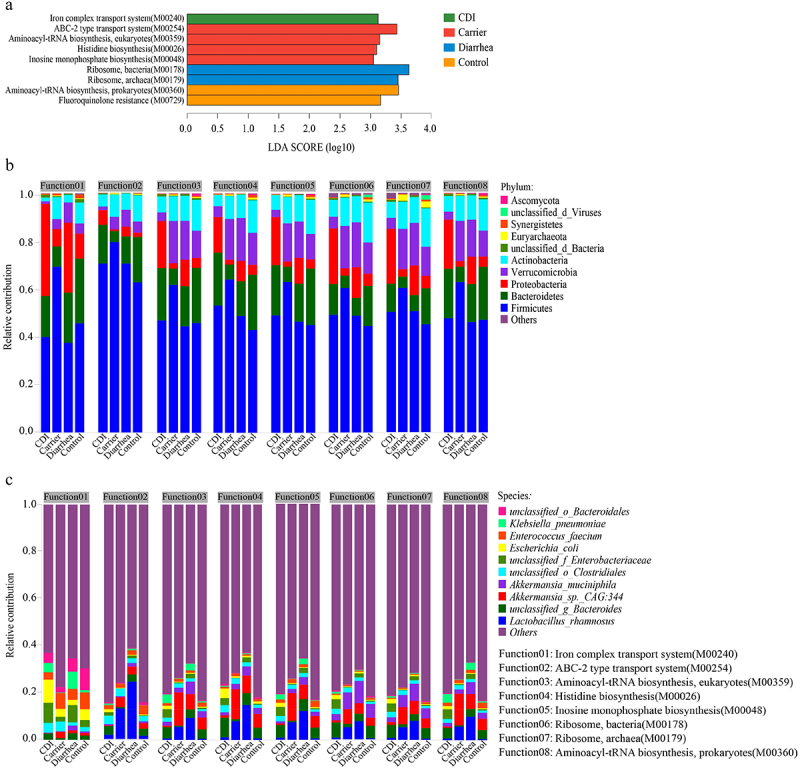
Different trends of functional modules from the fecal microbiomes of CDI, carrier, diarrhea and control participants. Linear discriminant analysis (LDA) effect size (LEfSe) is a tool to identify biomarkers from high dimensional data of two or more groups using. This tool clarifies about statistical significance and biological correlation and can identify statistically different biomarkers between groups. Identified biomarkers ranked by effect size, and only KEGG modules meeting an LDA significant threshold > 2.5 and adjusted *p* < 0.05 were shown (a). Relative contributions for modules to microbial phylum (b, top 10) or species (c, top 10) was estimated. Abbreviations: LDA, linear discriminant analysis; LEfSe, linear discriminant analysis effect size.

To further explore features of the potential functional consequences of microbial community in four cohorts, we compared the relative abundances of the potential functional pathway of gut microbiota at modules levels. The top 10 abundant potential functional modules in the fecal of four cohorts were ribosome (M00178 and M00179), aminoacyl-tRNA biosynthesis (M00360 and M00359), reductive pentose phosphate cycle (Calvin cycle, M00165 and M00167), glycolysis (M00001), ABC-2 type transport system (M00254), Gluconeogenesis (M00003) and membrane transport (M00258) (Supplementary Figure S8). We identified 9 modules that were differentially abundant (LEfSe: adjusted *p* < 0.05, LDA > 2.5) among four groups (Figure 4(a)). Module involved in iron complex transport system (M00240) was enriched in the CDI cohorts. We also observed significantly higher abundance of modules related to ABC-2 type transport system (M00254), aminoacyl-tRNA biosynthesis (M00359), histidine biosynthesis (M00026) and inosine monophosphate biosynthesis (M00048) in the Carrier. While, ribosome (M00178 and M00179) was enriched in the Diarrhea cohorts. On the contrary, fluoroquinolone resistance (M00729) and aminoacyl-tRNA biosynthesis (M00360) were particularly enriched in the Control cohorts. Using ANCOM, we identified 15 additional modules (etc. M00709: Macrolide resistance, MacAB-TolC transporter, M00696: Multidrug resistance, efflux pump AcrEF-TolC etc.) among the four groups (Supplementary Figure S9A), which were inconsistent with the results obtained from the LEfSe analysis. The contributions of phylum and species to modules differed among four cohorts (Figure 4(b,c)). The phylum Proteobacteria contributed to M00240 with increase abundances in CDI group than the other three groups, while the abundance of Verrucomicrobia and Actinobacteria were reduced (Figure 4(b)). Firmicutes contributed to M00254, M00359, M00026 and M00048 with increase abundances in Carrier group, while the abundance of Bacteroidetes and Proteobacteria were decrease in Carrier group (Figure 4(b)). The abundances of *unclassified_f_Enterobacteriaceae* and *Escherichia_coli* contributed to M00240 were relatively higher in CDI, while the abundances of *Lactobacillus_rhamnosus* and *Akkermansia_sp._CAG:344* contributed to M00254, M00359, M00026 and M00048 were relatively higher in Carrier (Figure 4(c)). Overall, these findings indicate a microbial community-level shift followed by an alteration in potential functional potential of the gut microbiota. Thus, pathway analysis is important for understanding the potential functional aspects of the microflora under pathological conditions, and these findings might provide insights into potential novel therapeutic targets.

### 3.6. Antibiotic resistance profiles

**Figure 5.**
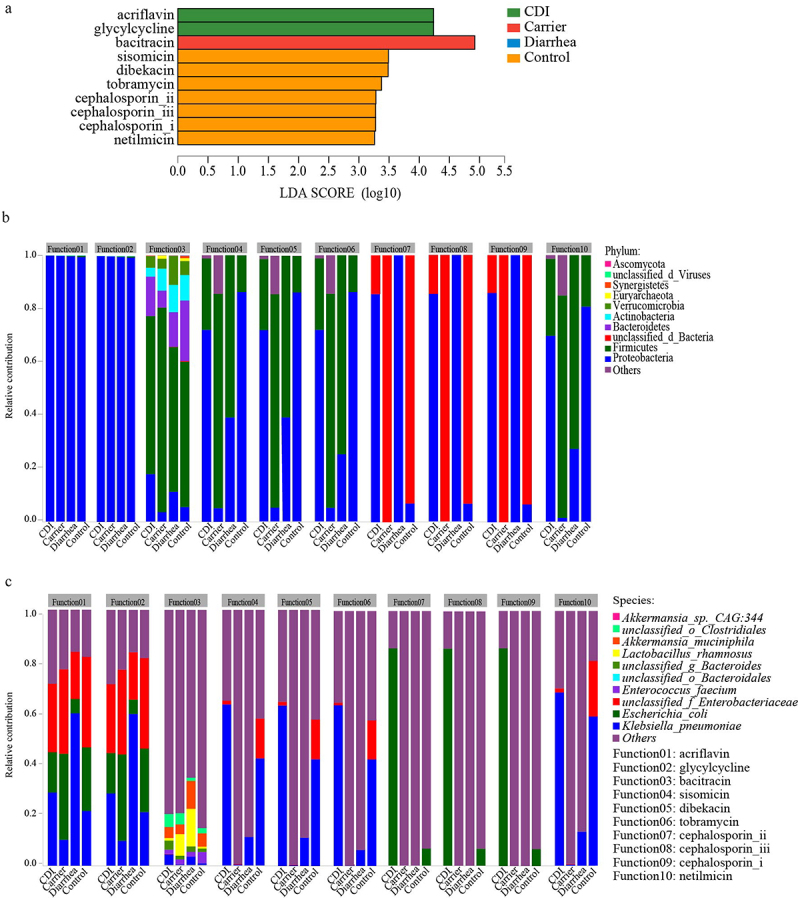
Different trends of antibiotic resistance gene (ARG) from the fecal microbiomes of CDI, carrier, diarrhea and control participants. LDA is a tool to identify biomarkers from high dimensional data of two or more groups using. This tool clarifies about statistical significance and biological correlation and can identify statistically different biomarkers between groups. Identified biomarkers ranked by effect size, and only taxa meeting an LDA significant threshold > 3.0 and adjusted *p* < 0.05 were shown (a). Relative contributions for ARG to microbial phylum (b, top 10) or species (c, top 10) was estimated. Abbreviations: LDA, linear discriminant analysis; ARG, antibiotic resistance gene.

The high concentrations of antibiotics used in clinical practice provide a strong selective pressure that favors the exchange of antibiotic resistance genes. To investigate the antibiotic resistance profile present in the gut microbiota of four cohorts, the unigenes identified in the metagenome data were screened for ARG using the Antibiotic Resistance Database (ARDB). A total of 535 ARGs were identified in ARDB. Among these ARGs, two resistance functions were enriched in CDI, including acriflavine and glycylcycline, resistance function of bacitracin was enriched in Carrier, and resistance functions of sisomicin, dibekacin, tobramycin, cephalosporin_ii, cephalosporin_iii, cephalosporin_i and netilmicin were enriched in Control (LDA > 3.0, adjusted *p* < 0.05; Figure 5(a)). Confirming the LEfSe findings, ANCOM analysis also enabled us to identify two resistance functions (bacitracin and cephalosporin) that were differentially abundant among the four groups (Supplementary Figure S9B). These detected ARGs were further analyzed for their microbial origin. In four cohorts, resistance function of acriflavine and glycylcycline were highly enriched in phylum Proteobacteria (>99%, Figure 5(b)). The phylum Firmicutes contributed to resistance function of bacitracin with increase abundances in Carrier group, while the abundance of Bacteroidetes and Proteobacteria were decrease in Carrier group (Figure 5(b)). The abundance of *Klebsiella_pneumoniae* contributing to resistance function of acriflavine and glycylcycline were relatively higher in CDI than in Carrier, but the abundances of *Escherichia_coli* and *unclassified_f_Enterobacteriaceae* were opposite (Figure 5(c)). The abundances of *Lactobacillus_rhamnosus* contributing to resistance function of bacitracin were relatively higher in Carrier than in CDI, while the abundances of *Klebsiella_pneumoniae* and *Escherichia_coli* were relatively higher in CDI (Figure 5(c)). Overall, the contributions of phylum and species to ARGs differed among the four cohorts (Figure 5(b,c)). These results indicate that different types of gut microbes contribute differently to the occurrence of antibiotic resistance genes and types.

### 3.7. Putative microbial correlations in each group

**Figure 6.**
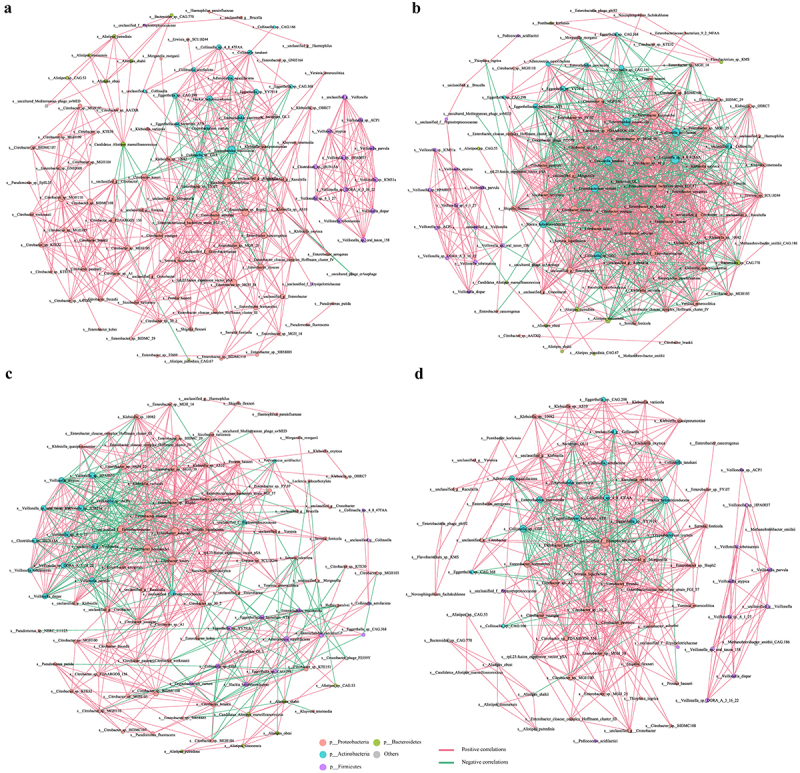
Network analysis from the fecal microbiomes of CDI (a), carrier (b), diarrhea (c) and control (d) participants. Each node represents species and is colored according to phylum-level taxonomy. Node size indicates relative abundance of a species, red lines indicate a positive association between nodes, and blue lines indicate a negative association between nodes. Line thickness indicates magnitude of the Spearman correlation coefficient, where thickness increases with magnitude.

Given the notably distinct microbiome composition among the four groups, we compared the topology of the microbial correlation networks (Figure 6). We found that the microbial correlation network of the CDI group has quite different structure compared to the other three groups. The overall microbial correlations in the CDI group are much more complex with more negative correlations than those in the other three groups (Figure 6). We also observed the disappearance of some correlations in CDI compared to other three cohorts. In order to quantify the difference of the network structure, we calculated the number of nodes, number of edges, average degree (the average number of connections per node) and clustering coefficient (measure of how complete the neighborhood of a node is, Supplementary Table S4). In general, the network of the CDI group has fewer edges and clustering coefficient, lower average degree, but higher nodes. These results suggest an altered microbiota relationship in CDI, where bacteria became more intertwined and less specialized, further implicating the importance of equilibrium among bacteria for human health.

## 4. Discussion

Our study provides a comprehensive analysis of human fecal microbiota and potential functional study in CDI, Carrier, Diarrhea and Control cohorts using a deep-level metagenomics. The novel finding here is that both specific gut microbiota species and their potential functions are involved in CDI. There were significant alterations in the alpha and beta diversity in the four cohorts. By further employing LEfSe and ANCOM analysis, we successfully identified several changes in the microbiota of CDI that could be further evaluated as predictive markers. Random forest analysis revealed that *Novosphingobium_fuchskuhlense* (CDI vs Carrier, Carrier vs Diarrhea), *unclassified_f_Peptostreptococcaceae* (CDI vs Diarrhea), *Flavobacterium_sp._KMS* (CDI vs Control) were the most important microbial features. The random forest classifier based on a combined set of features microbial can achieve a powerful diagnostic performance in differentiating four groups (AUC >0.91). In addition to the altered intestinal microbiome, we showed that a set of microbiome potential functions were significantly different among our four cohorts. Specific gut microbiota species and modules may provide a diagnostic potential to differentiate CDI subjects from Carrier, Diarrhea and Control groups.

In this study, the Chao1 index was higher for Control patients than CDI and Carrier patients, while no significant difference was found between CDI and Carrier. This is similar to our metagenomic approach, previous study using less accurate 16S rRNA sequencing, that found no differences in gut microbiota between CDI and Carrier.^25–27^ Decreased species abundance and diversity might be features of CDI but could also occur in many hospitalized patients without CDI, this could explain why no differences were found among CDI and Carrier cohorts in this study. Compared with those studies reporting associations between gut microbiota and CDI using 16S rRNA sequencing techniques,^14,28–31^ our study used metagenomic sequencing technique which provides higher resolution of the composition of the microbiota, containing abundance of bacteria at the species level and also information on sensitivity, specificity determinations, network analyses and direct implication of their metabolic pathways. Therefore, we believe that our findings are likely to be more accurate in clarifying the possible relationship between the gut microbiota and CDI. There are also two studies that have applied a metagenomics approach,^12,13^ which did find deviations in the gut microbiota. Interestingly, we first reported that Shannon index at modules level was higher in CDI than in Carrier and Control, this result clearly demonstrates that the change of intestinal microflora functions reflected the clinical cohorts. In our study, community PCoA highlighted species-level differences in microbiome composition between CDI and the other two groups. This is in accordance with a previous study which showed evident separation between the Control and CDI patients.^25^ These results indicate a significant global shift in gut microbiota and potential functions among our four cohorts.

In this study, the main phyla were Firmicutes, Bacteroidetes and Proteobacteria. The gut microbiota is emerging as a promising target for the management or prevention of inflammatory and metabolic disorders in humans. The human gut microbiota is mostly composed by two dominant bacterial phyla, Firmicutes and Bacteroidetes that represent more than 90% of the total community, and by other subdominant phyla including Proteobacteria, Actinobacteria, and Verrucomicrobia,^32–34^ this is in accordance with our study findings.

Both microbial LEfSe and ANCOM analysis successfully identified several changes in the microbiota of four cohorts. In accordance with our study, in CDI samples, the overrepresentation of opportunistic pathogens, such as *Escherichia_coli*,^35^ *Klebsiella* and members of *Enterobacteriaceae*,^13^ may simply reflect a “blooming” phenomenon due to a reduced ecological niche competition.^36^ However, different from our finding that *Bacteroides_massiliensis* was enriched in CDI cohort, *Bacteroides* have been shown to have a role in the resistance against CDI^37–40^ and are involved in carbohydrate metabolism and regulation of immune functions.^41–43^ In mice, *Bacteroides* was found to be one of the genera involved in the recovery of disease after bacteriotherapy using a mix of bacteria.^44^ Therefore, further studies should be carried out to understand the interaction between *Bacteroides massiliensis* and *C. difficile* colonization/infection. Meta-analyses of randomized controlled trials support the efficacy of the probiotics *Lactobacillus rhamnosus*, *Saccharomyces boulardii* and *Lactobacillus rhamnosus* GG in the prevention of CDI.^45^ This fits our result that *Lactobacillus rhamnosus* was enriched in Diarrhea group who had tested NAAT negative on clinical *C. difficile* testing, indicating that these bacteria may play a role in preventing or treating CDI colonization and development. The present study is the first to report increased abundance of *Novosphingobium_fuchskuhlense* in Carrier/Diarrhea/Control compared with CDI. Thus, *Novosphingobium_fuchskuhlense* could perhaps play a protective role in patients at risk for CDI. Furthermore, our classification analysis revealed that *Novosphingobium_fuchskuhlense* achieved a powerful classification potential for distinguishing CDI from Carrier/Diarrhea/Control.

In this study, we found that the overall microbial correlations in the CDI group are much more complicated with more negative correlations, reduced edge counts, lower clustering coefficients, decreased average degrees, and an increased number of nodes than those in other three groups, and we also observed the disappearance of some correlations in CDI compared to the other three cohorts. The increased number of nodes combined with fewer edges in the CDI cohort suggested that CDI being a state in which physiological microbial correlations are disrupted where cooperative interactions are significantly reduced. This reduction in connectivity and the clustering coefficient points to a breakdown in microbial alliances that are essential for a resilient microbiome structure. Such alliances typically stabilize microbial ecosystems and protect against pathogenic colonization.^27^ In CDI, however, this weakened network may permit greater ecological opportunities for opportunistic pathogens like *C. difficile* to colonize,^13^ as niches normally occupied by beneficial microbes are vacated due to the depletion of key microbial biomarkers. Furthermore, the higher prevalence of negative correlations in the CDI network suggests an increase in competitive interactions, which could result from the competitive pressures within a disrupted microbiome. This shift from cooperative to competitive interactions may create an ecosystem with less functional redundancy and specialization. As indicated by the reduced average degree in the CDI network, a loss of microbial specialization may occur, with distinct taxa unable to perform unique functional roles.^27^ This reduced functional diversity could impair the microbiome’s ability to support critical host functions, including nutrient metabolism and immune modulation, thus weakening the host’s resilience to CDI and other pathogens.^13^ These findings collectively suggest that CDI disrupts not only microbial composition but also microbial interaction patterns, shifting the microbiome from a cooperative to a competitive network state. This altered network topology indicates a state of microbial imbalance that may mediate disease susceptibility. Further studies should be required to clarify the directionality of these interactions and to assess how restoring these microbial correlations could enhance the gut microbiota’s resilience against CDI.

In this study, modules involved in iron complex transport system (M00240) were enriched in CDI. Nowadays, bacterial antibiotic resistance is becoming a global challenge. Resistance to antibiotics makes the treatment of bacterial infectious diseases difficult. Antibiotic resistance of bacteria spreads through the acquisition of resistance genes, which exist in transposons, integrons, and plasmids.^46^ The ability of *C. difficile* for getting mobile genetic elements (e.g. from *Enterococci*) and creating resistance to the most powerful antibiotics shows the importance of transposons.^47^ Proteins containing iron, [Fe-S]-clusters and iron-coordinated heme are indispensable for the bacterial metabolism. Consequently, iron is an essential micronutrient for the growth of most bacteria including *C. difficile*.^48^ For bacteria, classical iron transport systems consist of an outer membrane receptor, a periplasmic binding protein, and an inner membrane ABC transporter, which work in concert to deliver iron from the cell surface to the cytoplasm.^49^ Thus, the increased level of iron complex transport system might contribute to an improvement in intestinal CDI.

Modules related to ABC-2 type transport system (M00254), aminoacyl-tRNA biosynthesis (M00359), histidine biosynthesis (M00026) and inosine monophosphate biosynthesis (M00048) were enriched in Carrier. ABC type transporters play a role in the import or export of a considerable number of substrates, ranging from ions to macromolecules, and in many pathogens play a role in physiological functions that include nutrient transport, signal transduction, export of virulence factors and antibiotic resistance.^50^ Previous study indicated that the differential expression of ABC transporters could play a role in the virulence of *C. difficile* by improving the import or export of essential molecules involved in adaptive features of the bacterial cell and therefore in bacterial virulence.^51^ Previous study also revealed that the ABC transporter from *C. difficile* confers resistance against multiple antibiotics such as nisin and gallidermin.^52^ Of note, ABC type transport system were enriched in Carrier in this study.

In our study, a range of antibiotic resistance categories were also identified in the gut microbiota of the four clinical cohorts. Based on the analysis of the origin, ARGs profiles were found to be correlated with microbial community compositions. We found that two resistance functions were enriched in CDI, including acriflavine and glycylcycline, resistance function of bacitracin was enriched in Carrier, and resistance functions of sisomicin, dibekacin, tobramycin, cephalosporin_ii, cephalosporin_iii, cephalosporin_i and netilmicin were enriched in Control. In congruence with our data, it has been previously shown that bacitracin direct inhibited the activity of *C. difficile* and protected stem cell-derived human intestinal organoids as well as human gut epithelial cells from intoxication with TcdB.^53–55^ Tigecycline, the first licensed broad-spectrum glycylcycline antibiotic against both Gram-positive and Gram-negative facultative and obligate anaerobes, and tigecycline might have a relatively low propensity to promote CDI in comparison to other broad-spectrum antibiotics.^56^ Previous study revealed that tigecycline exposure did not induce *C. difficile* proliferation or cytotoxin production despite reduced competing microflora in human gut.^57^ Tigecycline inhibited establishment of colonization, but did not reduce concentrations of *C. difficile* once high-density colonization was established in mice.^58,59^ Tigecycline also inhibits protein synthesis and has been shown to inhibit *C. difficile* toxin production and sporulation in mice.^60^ Thus, tigecycline may offer physicians an alternative treatment for CDI. There is no study referring to acriflavine in CDI, thus more studies are needed to assess the utility of this antibiotic in the treatment of CDI. Due to application of antibiotics in clinical patients, the widespread presence of antibiotic resistance genes and antibiotic resistant bacteria were also found in the CDI cohort. Further investigation is needed to fully define the mechanisms of antibiotic resistance in *C. difficile*. Identification and characterization of any additional antibiotic resistance mechanisms may aid in preventing CDI in the future.

In our study, species and potential functional contribution analyses at the phylum and species levels were employed to evaluate the detailed correlation between the relative abundance of microbiota, modules and influenced ABGs family. These results indicated that high application of antibiotics might enable the spread of antibiotic resistant genes and bacteria. Previous study also revealed that administration of antibiotics to premature infants after the first week of life can prolong the enrichment of the resistome.^61^ Thus, it is necessary to reduce potential environmental contamination risks associated with antibiotic resistance from feces of clinical patients and the abuse of antibiotics. In this study, the abundances of *Klebsiella_* belong to phylum Proteobacteria contributed to resistance function of acriflavine and glycylcycline which were relatively higher in CDI than in the other three cohorts. Previous studies have revealed that increased relative abundance of Proteobacteria is associated with CDI and has been proposed to be a risk factor.^8,26,62,63^ In ARGs, the use of clindamycin or fluoroquinolones increases efflux pump protein expression, which confers resistance to acriflavine, aminoglycosides, glycylcycline, macrolides and β-lactams. This protein was primarily detected in Proteobacteria, which showed a relatively abundant increase under treatment.^64^ Additionally, in cases where antibiotic administration is required, the route of antibiotic administration should be considered carefully when different options are available. The results act as a cautionary insight into the prevalence of antibiotic resistance in different cohorts, while the mechanisms governing this phenomenon of antibiotic resistant variability are currently being explored in our laboratory.

Recent advances in molecular ecological techniques, such as metagenomics, have enabled a more efficient pathway to address the taxonomic and potential functional composition of gut microbiota. The 16S rRNA sequencing may not have captured additional insights associated with the disease status available at the species or strain level. Metagenomics methods might be quite useful to identify consistent biological signatures (species and module) to discover potential novel diagnostics and therapeutic targets of CDI. We acknowledge that our study also presents some limitations. This study could not assess the cause–effect relationship between specific alterations of the microbiota and clinical status. Even though our analysis was data-driven, it requires further validation. Future longitudinal or prospective studies may provide further evidence to support our hypotheses. This study also failed to address the potential confounding impact of antibiotics. Previous studies have shown that taxonomic and potential functional alterations induced by strong antibiotic usage are similar to alterations observed in CDI patients, but the impact can be decoupled.^65^ In future studies, the antibiotic confounding impact was also taken into account. Sequencing allows us to indicate potential coloization by identifying microbial patterns associated with *C. difficile*, but it does not provide definitive proof of colonization. Thus, more conclusive methods, such as culture-based techniques or other direct identification methods, would be necessary to confirm colonization. Most microbiome analyses to date have focused on metagenomic sequencing of bacteria in stool samples, but emerging research suggests that viruses, prokaryotes, and small molecules can also meaningfully impact health and disease.^1,66^ Meanwhile, multi-omics (such as metatranscriptomic and metabolomics) offer valuable insights into the functional aspects of the microbiome and its role in CDI. Therefore, future studies should consider a multi-omics and multi-kingdom approach to better evaluate factors influencing CDI.

## 5. Conclusion

In summary, we used metagenomics to gain an insight into both the compositional (profiles of microbiota) and the potential functional capabilities (KEGG modules and ARGs) alterations with patients’ diseases (*C. difficile* colonization and diarrhea) and controls. By comparing the gut metagenomes of these four cohorts, we identified both substantial overlap and differences in microbial composition and potential function among the four cohorts. Although it remains unclear to what extent these changes were determined by *C. difficile*, these results substantially increased our knowledge of the CDI gut microbiome. To determine if the microbiome is indeed involved in CDI, further follow-up prospective studies are required. Further studies integrating the omics data, that is, metatranscriptomics, proteomics, and metabolomics data would provide more insights into the potential function of the gut microbiota in the regulation of intestinal metabolism. To our knowledge, this is the first report on microbiome alterations using metagenomics data analysis among four cohorts. The present findings may be used to complement a potential diagnostic method for to discriminate CDI from Carrier, Diarrhea and Control. By incorporating these microbial profiles, future diagnostic tools could more accurately differentiate CDI from other types of disease states, leading to more effective interventions.

## Supplementary Material

Supplementary tables and figures.docx

Supplementary methods.docx

## Data Availability

Sequencing data generated from shotgun metagenomes in this study have been deposited with the NCBI SRA (PRJNA1067975) and are publicly available.
