# Supplementary material for: Metagenomic analysis reveals distinct patterns of gut microbiota features with diversified functions in C. difficile infection (CDI), asymptomatic carriage and non-CDI diarrhea
Source: Gut Microbes. 2025 May 14;17(1):2505269. doi: 10.1080/19490976.2025.2505269 (PMC12080279; doi:10.1080/19490976.2025.2505269)
Supplement: Supplementary tables and figures.docx [file KGMI_A_2505269_SM2009.docx]

**Supplementary tables**

**Table S1. Demographic characteristics of** **the enrolled subjects.**

| **Characteristics** | **CDI**  **(n = 47)** | **Carrier**  **(n = 17)** | **Diarrhea**  **(n = 14)** | **Control**  **(n = 26)** | ***P* value** |
| --- | --- | --- | --- | --- | --- |
| **Diarrhea** | Yes | No | Yes | No |  |
| **TcdB NAAT** | Yes | Yes | No | No |  |
| **Sex** |  |  |  |  | 0.241^#^ |
| Female | 26 (55.32) | 10 (58.82) | 8 (57.14) | 9 (34.62) |  |
| Male | 21 (44.68) | 7 (41.18) | 6 (42.86) | 17 (65.38) |  |
| **Age, Avg ± SD** | 65±16 | 56±19 | 63±16 | 63±13 | 0.290^*^ |
| **Age, n (%)** |  |  |  |  | 0.691^#^ |
| Aged > 65 | 26 (55.32) | 7 (41.18) | 6 (42.86) | 12 (46.15) |  |
| Aged < 65 | 21 (44.68) | 10 (58.82) | 8 (57.14) | 14 (53.85) |  |
| **Race, n (%)** |  |  |  |  | 0.385^#^ |
| White | 37 (78.72) | 11 (64.71) | 9 (64.29) | 16 (61.54) |  |
| Others/unknown | 10 (21.28) | 6 (35.29) | 5 (35.71) | 10 (38.46) |  |
| **Ethnicity, n (%)** |  |  |  |  | 0.159^#^ |
| Hispanic | 3 (6.38) | 0 (0.00) | 2 (14.29) | 0 (0.00) |  |
| Non-Hispanic/ Unknown | 44 (93.62) | 17 (100.00) | 12 (85.71) | 26 (100.00) |  |

^*^ One-way ANOVA, ^#^ Chi square test.

**Table S2. Permutational multivariate analysis of variance (PERMANOVA) in** **fecal microbiomes compositions.**

| **Characteristics** | **F** | **R^2^** | **Adjust *P*-value** |
| --- | --- | --- | --- |
| **Cohorts** | 2.079 | 0.059 | 0.004 |
| **Sex** | 1.559 | 0.015 | 0.064 |
| **Age** | 1.347 | 0.013 | 0.133 |
| **Race** | 0.987 | 0.010 | 0.458 |
| **Ethnicity** | 0.914 | 0.009 | 0.558 |

Race: White and others/unknown. Ethnicity: Hispanic, Non-Hispanic/Unknown. Here F represents the F-statistic: a larger F value indicate that the between-group variation is greater than within-group variation. R^2^ represents the variation explained by the model. *P* represents the adjust *P*-value calculated from permutation.

**Table S3. Generated data of the enrolled subjects**

| Indicators/Groups | CDI (n=47) | Carrier (n=17) | Diarrhea (n=14) | Control (n=26) | Total (n=104) | *P*-value |
| --- | --- | --- | --- | --- | --- | --- |
| Read length (bp) | 150 | 150 | 150 | 150 | 150 | - |
| Raw reads | 61473006±21692249 | 70571153±5062997 | 73994100±2007188 | 70208037±13948798 | 69061574±8920445 | 0.026 |
| Raw base (bp) | 9220951034±3253837495 | 10585673047±759449696 | 11099115021±301078271 | 10531205573±2092319822 | 10359236169±1338066835 | 0.026 |
| Clean reads | 61281565±21433443 | 70398098±4883351 | 73789138±1964033 | 70093286±13657569 | 66658410±16846527 | 0.025 |
| Clean base (bp) | 9187715556±3218419934 | 10556548489±733367226 | 11066016404±295116760 | 10511238761±2050192216 | 99951960865±2529563033 | 0.058 |
| Percent of clean reads in raw reads (%) | 99.51±0.91 | 99.76±0.15 | 99.72±0.42 | 99.82±0.16 | 99.68±0.35 | 0.199 |
| Percent of clean bases in raw bases (%) | 99.33±1.27 | 99.73±0.14 | 99.70±0.42 | 99.77±0.26 | 99.63±0.51 | 0.139 |
| Optimized reads | 57861821±21760526 | 66718932±6669469 | 70655532±2969003 | 66195798±13719154 | 65358021±8288960 | 0.029 |
| Optimized bases (bp) | 8677773514±3263531273 | 10005730922±1000942026 | 10596563376±445599118 | 9927726160±2057583510 | 9801948493±1242904944 | 0.029 |
| Percent of optimized reads in raw reads (%) | 89.30±17.57 | 94.39±3.64 | 95.46±2.13 | 93.50±5.99 | 93.16±7.01 | 0.234 |
| Percent of optimized bases in raw bases (%) | 89.28±17.57 | 94.37±3.65 | 95.45±2.13 | 93.48±5.99 | 93.14±7.00 | 0.234 |

**Table S4. Characteristics of microbial correlation networks associated with different groups.**

| **Groups** | **Edges** | **Nodes** | **Average degree** | **Clustering coefficient** |
| --- | --- | --- | --- | --- |
| **CDI** | 590 | 114 | 10.44 | 0.108 |
| **Carrier** | 1172 | 105 | 22.54 | 0.095 |
| **Diarrhea** | 701 | 107 | 13.23 | 0.111 |
| **Control** | 493 | 82 | 12.17 | 0.132 |

**Supplementary Figures**


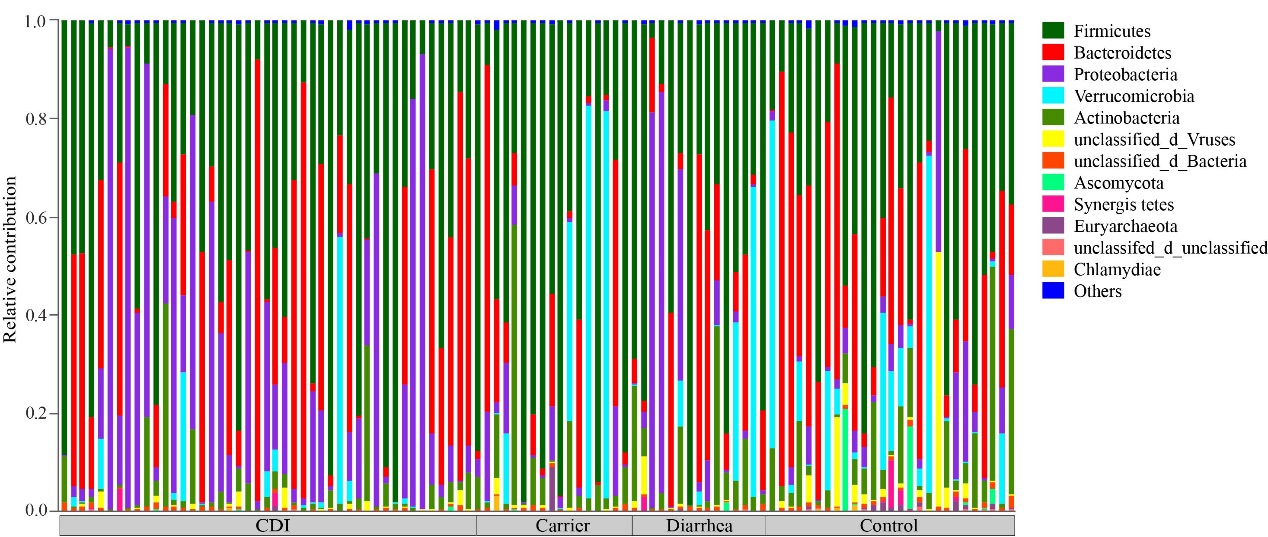


**Fig. S1** Phylum profiles from the fecal microbiomes of CDI, Carrier, Diarrhea and Control participants. Only top 12 phylum abundance in at least one sample were depicted. Otherwise, they were included in the category “others”. Each column represents one sample and each color represents one phylum.

**
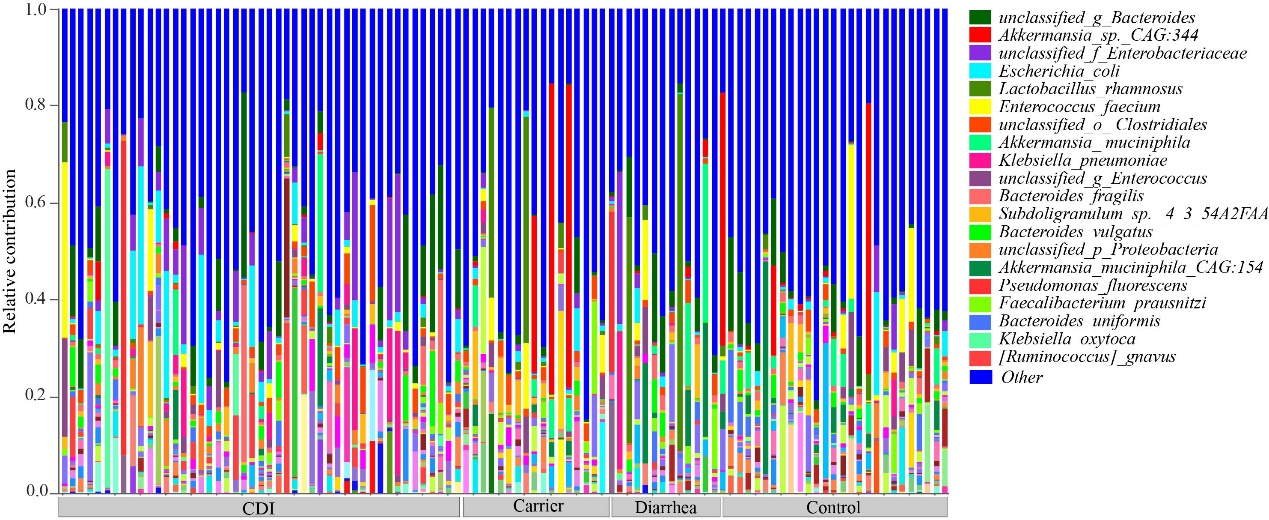
**

**Fig. S2** Species profiles in CDI, Carrier, Diarrhea and Control participants. Only top 20 species abundance in at least one sample were depicted. Otherwise, they were included in the category “others”. Each column represents one sample and each color represents one species.


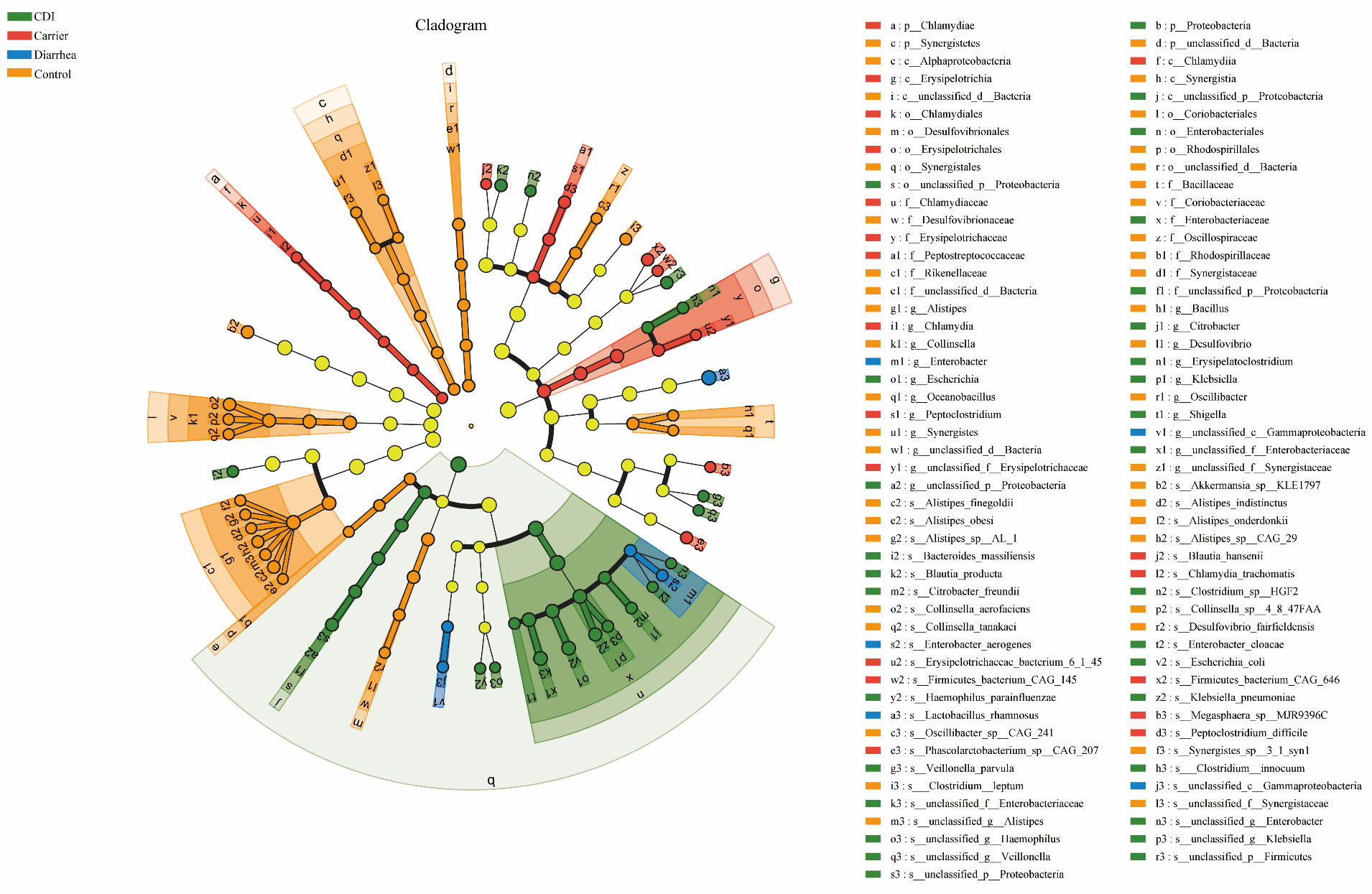


**Fig. S3** Cladogram of fecal microbial structure and predominant bacteria among CDI, Carrier, Diarrhea and Control participants. Cladogram representing the taxonomic hierarchical structure of the biomarkers identified between two cohorts. Each filled circle represents one biomarker.


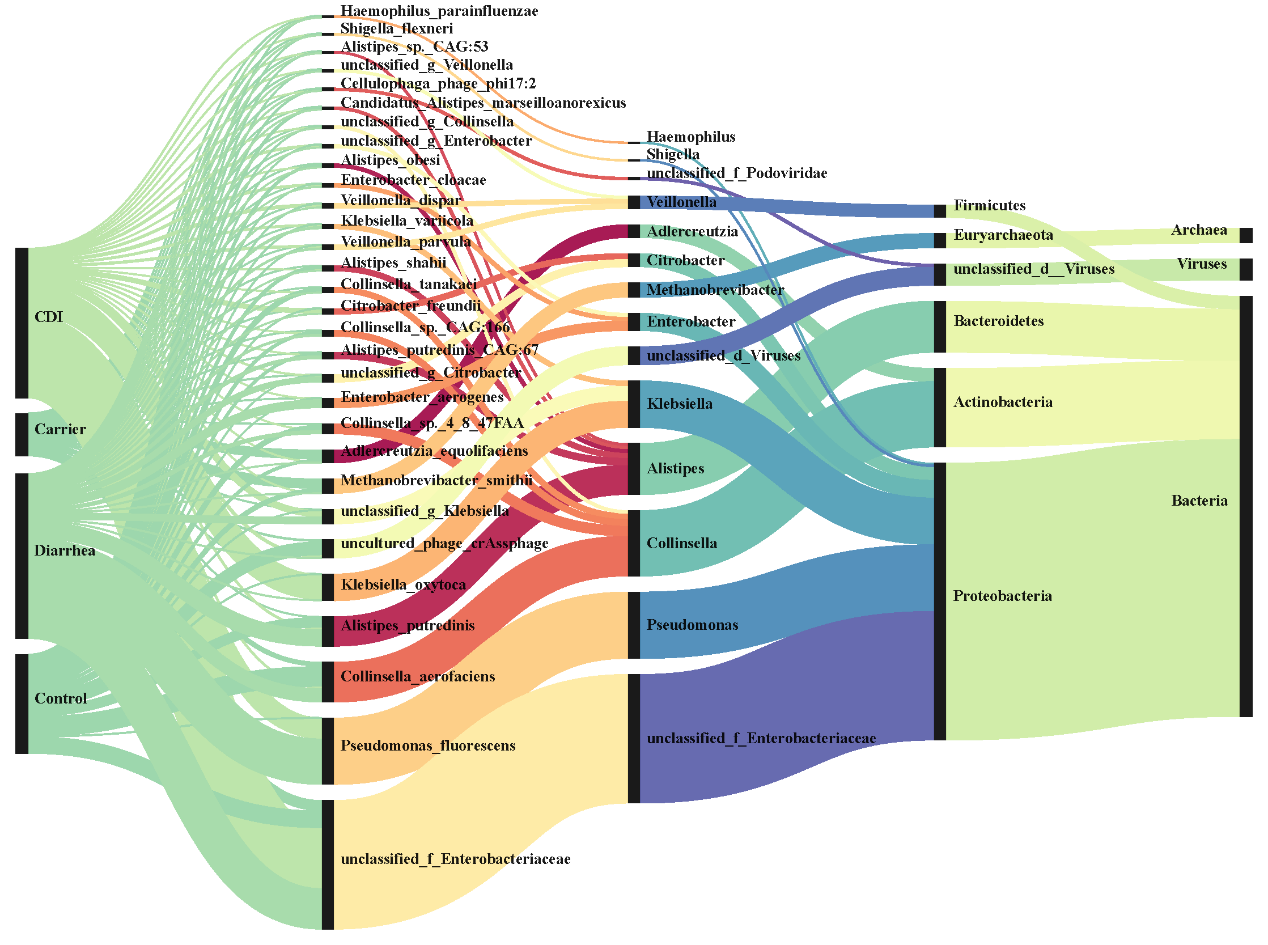


**Fig. S4** Sankey diagram showing the relative abundances of differentially abundant species identified by ANCOM in comparing CDI, Carrier, Diarrhea and Control participants. From left to right, names refer to cohorts, species, genera, phyla and kingdom.


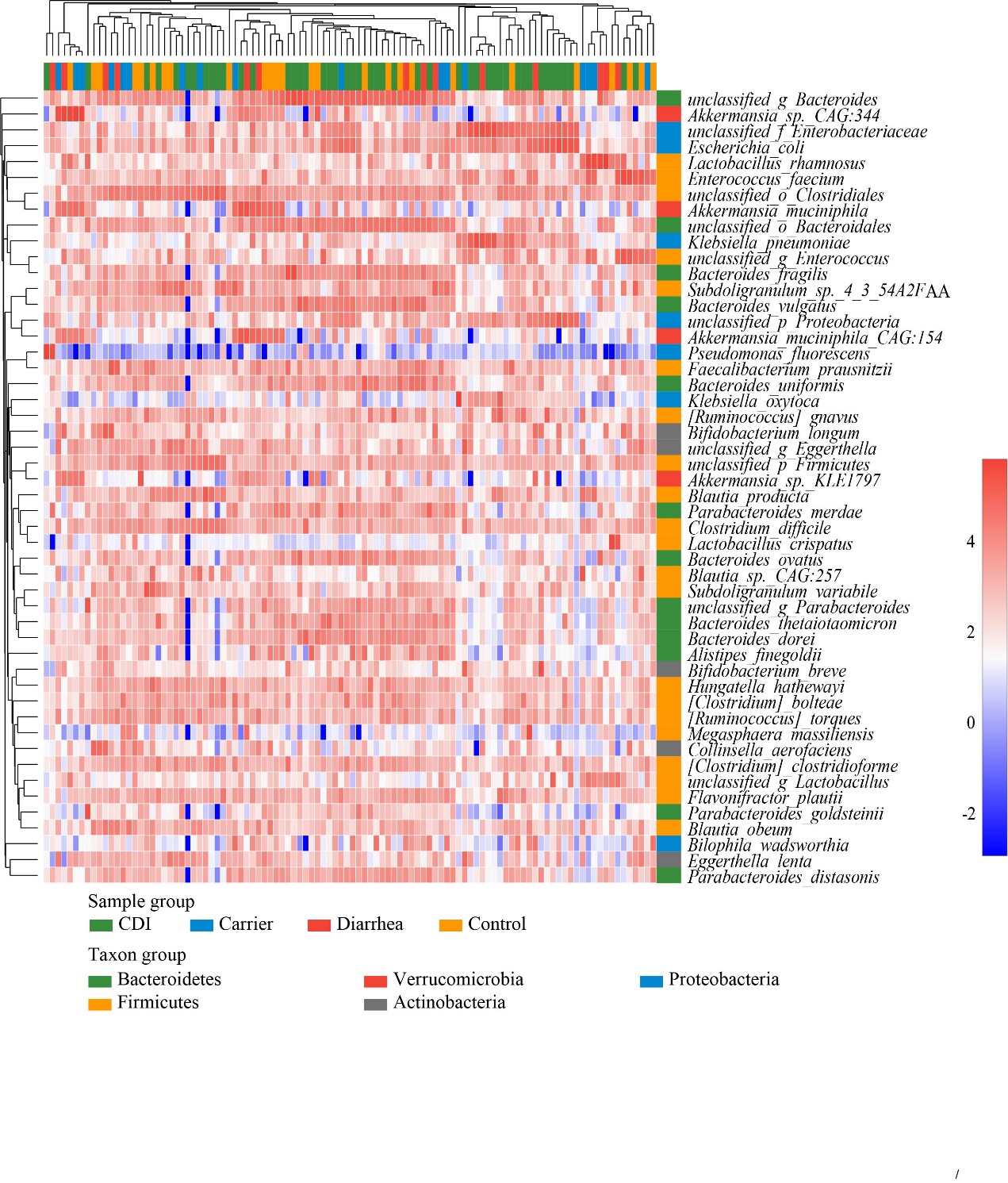


**Fig. S5** Relative abundance of top 50 species in CDI, Carrier, Diarrhea and Control participants. Heatmap representation of taxonomy abundance of the first 50 dominant species (ordinate) in all samples (abscissa). Relative values are color-coded and shown on the right side of the panel, ranging from blue (low abundance) to red (high abundance).

**Fig. S6** Classification analyses based on random forest models. The performance of classifier is measured by the AUCs. The dashed line is the random prediction that corresponds to an AUC of 0.5. On the abscissa the False Positive Rate (1-specificity) and on the ordinate the True Positive Rate (Sensitivity).

**
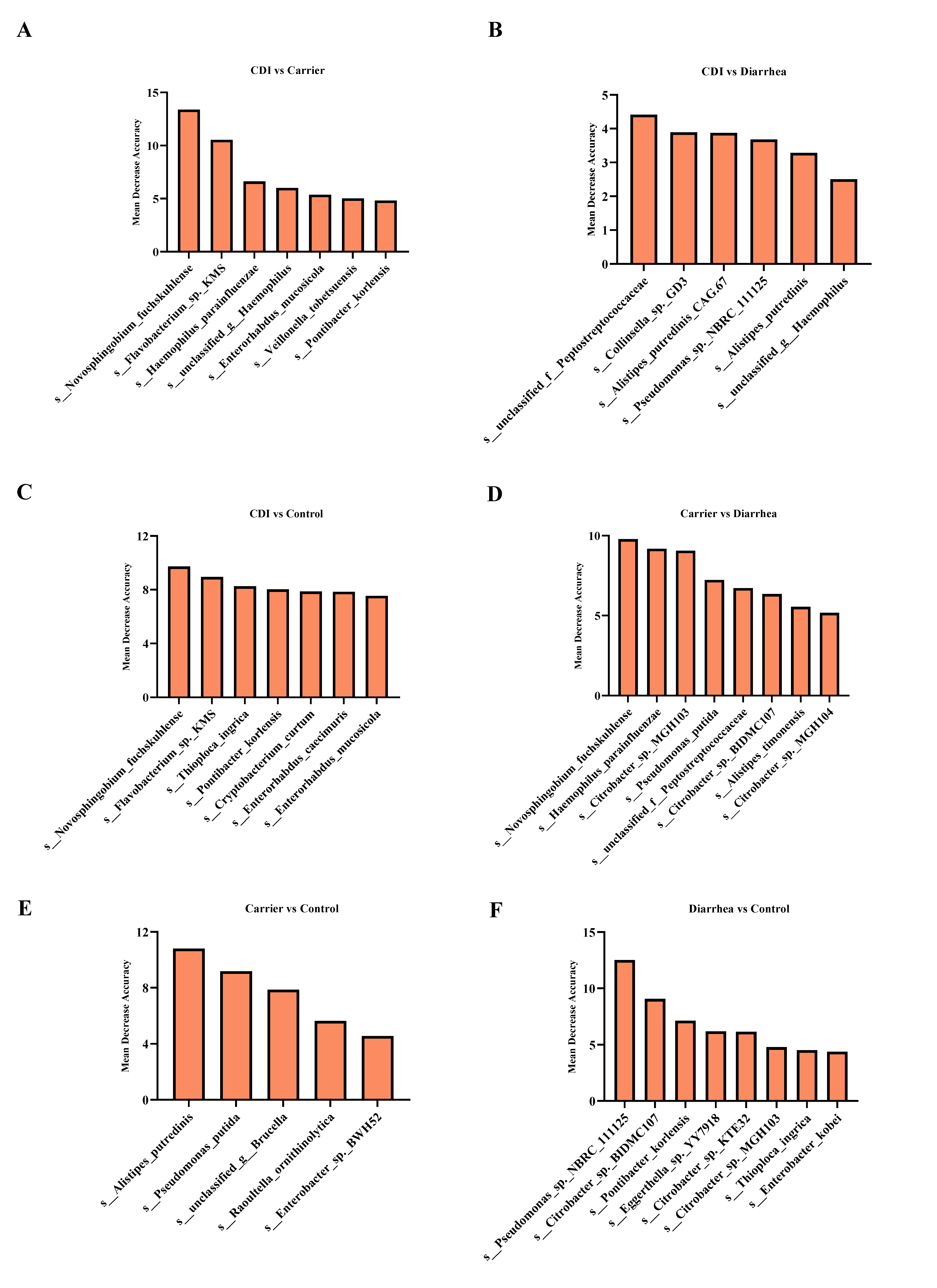
**

**Fig. S7** The top features ranked based on mean decrease accuracy. The lengths of the bars in the histogram represent the mean decrease accuracy, which indicates the importance of features for classification.

**
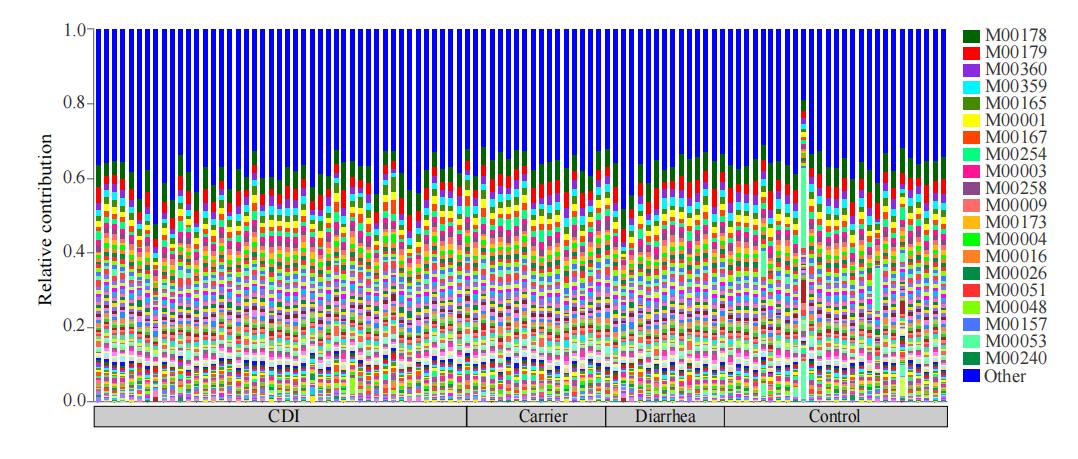
**

**Fig. S8** Modules profiles in CDI, Carrier, Diarrhea and Control participants. Only top 20 Modules abundance in at least one sample were depicted. Otherwise, they were included in the category “others”. Each column represents one sample and each color represents one module.


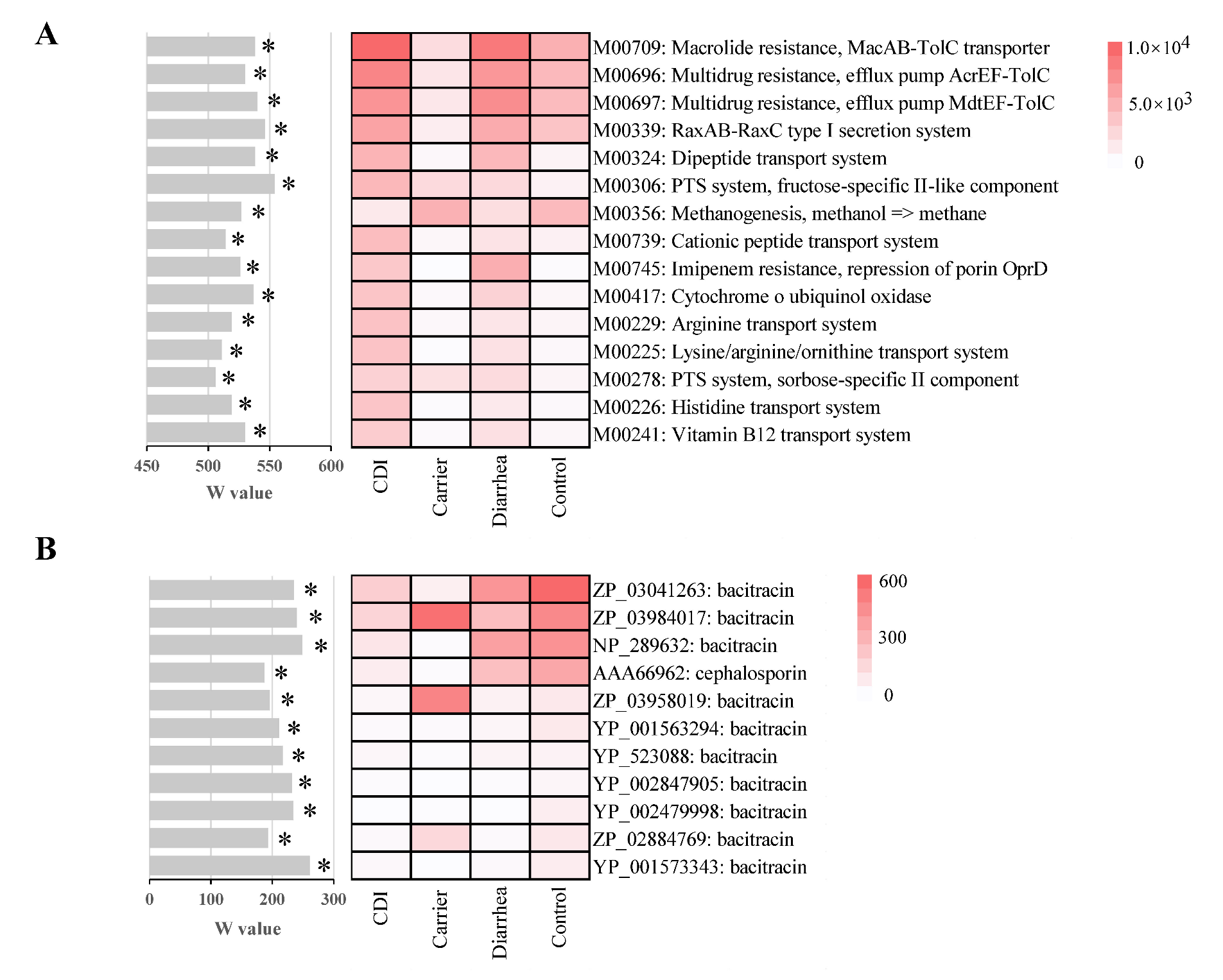


**Fig. S9** Relative abundances of differentially abundant modules (A) and antibiotic resistance (B) identified by ANCOM in comparing CDI, Carrier, Diarrhea and Control participants.
