# Supplementary material for: Metagenomic analysis reveals distinct patterns of gut microbiota features with diversified functions in C. difficile infection (CDI), asymptomatic carriage and non-CDI diarrhea
Source: Gut Microbes. 2025 May 14;17(1):2505269. doi: 10.1080/19490976.2025.2505269 (PMC12080279; doi:10.1080/19490976.2025.2505269)
Supplement: Supplementary methods.docx [file KGMI_A_2505269_SM2008.docx]

**SUPPLEMENTARY METHODS**

**Inclusion/exclusion criteria**

For the CDI and Diarrhea cohorts, patients with any of the following were excluded: (i) any doubt in regards to diarrhea classification, (ii) any CDI treatment for longer than 48 h before stool sample collection, (iii) presence of colostomy, and (iv) known diagnosis of Inflammatory Bowel Disease. Any patients with the following were excluded from Carrier and Control cohorts: (i) ≥2 loose stools in 24 h preceding enrollment, (ii) history of CDI in past 6 months, (iii) received any CDI treatment for >24 h or (iv) were tested for CDI in the 7 days preceding enrollment, and (v) known diagnosis of Inflammatory Bowel Disease. Stool was aliquoted within 72 h of production by the patient and stored at -80◦C.

**DNA extraction, shotgun metagenomic sequencing, and quality control**

For DNA extraction, fecal samples (200 mg) were performed using Mag-Bind® Universal Metagenomics Kit (Product# M5633-01, Omega Biotek) and DNeasy PowerSoil Kit (Catalog# 12888-100, Qiagen) according to manufacturer's instructions. Quality of the extracted DNA was measured by 1% agarose gel electrophoresis and Qubit® 3.0 Fluorometer (ThermoFisher). The extracted DNAs were then used for shotgun metagenomic library construction. Sequencing was then performed on the Illumina HiSeq X Ten platform, and 150 bp paired-end library was constructed for each sample. The quality of the library was examined by Agilent 2100 bioanalyzer.

**Reads quality filtering and de novo assembly**

The sequence quality filtering process involved several steps and started by removing reads that contained 10% or more ambiguous bases. The adapter sequences were removed (15 bases or longer sequence aligned to the adapter sequence) by fastp (V0.20.0). We continued the filtering process by applying a read length and average quality cut-off. The reads of less than 50 bp in length and an average quality score of less than 20 were removed ^1^. Furthermore, a filtering step is added to remove the sequence of the human genome so as to reduce the interference of the host sequence on the subsequent analysis using SOAP2 ^2^. The parameters were as follows: identity greater than or equal to 90%, -l 30, -v 7, -M 4, -m 200, -x 400 ^3^. Then, the high-quality reads of each sample were assembled by the SOAPdenovo software (V2.04), with the parameters -d 1, -M 3, -R, -u, -F, -K 55 ^4^. After de novo assembly for each sample independently, all reads that not used from all samples were combined and performed mixed assembly in order to maximize the usage of data. Subsequently, we broke the assembled Scaffolds from N connection and obtained the Scaftigs. At last, the fragments longer than 500 bp in all Scaftigs were used for further analysis ^5^.

**Gene prediction and construction of the nonredundant gene set**

We used MetaGeneMark (V2.10, http://topaz.gatech.edu/GeneMark/) to predict open reading frames (ORFs) from the Scaftigs assembled from each sample as well as the Scaftigs from the mixed assembly ^6^. Then, those ORFs with length <100 bp were filtered out. For the predicted ORFs, CD-HIT software (V4.6.1, http://www.bioinformatics.org/cd-hit) was used to reduce sequence redundancy ^7^ and the unique initial gene catalogue (the genes here refer to the nucleotide sequences coded by unique and continuous genes) was obtained. To obtain the gene catalogue (unigenes) eventually used for subsequently analysis, the clean data of each sample were mapped to the unique initial gene catalogue using Bowtie2 (V2.3.4.1). The Bowtie2 parameters for paired-end reads are listed below: -p8 -very-sensitive-local -k 100 -score-min L, 0, 1, 2. Based on the number of mapped reads and the length of gene, the abundance information of each Unigene in each sample was statistically analyzed ^8^.

**Functional gene annotation**

Functional annotation of metagenomes was conducted using DIAMOND software (V2.0.13, http://www.diamondsearch.org/index.php) to blast unigenes to the NCBI non-redundant protein database (nr, ftp://ftp.ncbi.nih.gov/blast/db/), Kyoto Encyclopedia of Genes and Genomes (KEGG) database (http://www.kegg.jp/kegg/), the antibiotic resistance genes database (ARDB, http://ardb.cbcb.umd.edu/) and the pathogen-host interactions database (http://www.phi-base.org) with the parameter setting of blastp (e-value cutoff of 1e^-5^) ^5, 9^. For each sequence's blast result, the best Blast Hit was used for subsequent analysis. The relative abundance of each functional hierarchy equaled the sum of relative abundance annotated to that functional level.

**Taxonomic assignment of genes, construction of taxonomy and profiles of relative abundance**

The output files of nr BLAST were analyzed using MEGAN (V4.6, http://ab.inf.uni-tuebingen.de/software/megan4/) ^10^. The software reads the results of a BLAST comparison as input and attempts to place each read on a node in the NCBI taxonomy using the lowest common ancestor algorithm. The NCBI taxonomy is displayed as a tree, and the size of each node is scaled to show how many reads are assigned to the corresponding taxon. Then, the relative abundance of each taxonomic level was summed from the same taxonomy ^1^. The relative abundances of all taxonomic groups were used to generate taxonomy-relative abundance profiles of all samples.

**Computation of relative gene abundance**

Reads mapping to multiple genes were reassigned to a "most likely" gene using PathoScope (V1.0) ^11^, which uses a Bayesian framework to examine each read's sequence and mapping quality within the context of a global reassignment ^1^.

**Alpha and beta diversity**

Alpha diversity (within-sample diversity) was quantified by the Chao1 (abundance based estimator of taxa richness) and Shannon (estimator of taxa richness and evenness: more weight on richness) index using the relative abundance profiles at species, modules or antibiotic resistances levels ^12^. High alpha diversity indicates high diversity of gut microbiota, modules or antibiotic resistances within a sample. Beta diversity (between-sample diversity) analyses including principal coordinates analysis (PCoA) tests for relative similarities of microbial communities were generated from a Bray-Curtis dissimilarity values based on the relative abundances of species, modules or antibiotic resistances and R vegan package (V2.4.3) was used for plotting.

**Clustering analysis**

For clustering analysis of the species-level bacteria compositions, the average-linkage hierarchical clustering algorithm by R function "hclust" (V3.6.3) was applied to cluster stool samples and species based on their similarities. A heatmap figure was generated based on the clustering result by the "gplots" package of R package (V3.1.3).

**Linear discriminant analysis effect size algorithm**

The linear discriminant analysis (LDA) effect size (LEfSe) algorithm was then performed on the Huttenhower lab Galaxy server (http://huttenhower.sph.harvard.edu/lefse/) by importing the species relative abundance or function pathway values and associated sample metadata (V2.0.13) ^13^. LEfSe first uses non-parametric factorial Kruskal-Wallis sum rank test to find significantly and differentially abundant features, then the Wilcoxon rank sum test to ensure the identified feature is biologically relevant. LDA is then performed on the identified features to determine the log10 effect size of each differentially significantly abundant feature. The threshold used to consider a discriminative feature for the logarithmic LDA score was set to >3.0 for different trends of species or antibiotic resistance and >2.5 for different trends of modules. The false discovery rate (Benjamini-Hochberg FDR) method was used to adjust the *P* values for multiple test correction.

**ANCOM analysis**

Differential abundance analysis was conducted at the species, modules or antibiotic resistances levels using ANCOM (analysis of composition of microbiomes) implemented in the QIIME2 pipeline (V2022.2) ^14^. ANCOM compares the relative abundance of taxa among multiple groups by log-ratio of the abundance of each taxon to the abundance of all the remaining taxa one at a time. ANCOM was conducted after removing spurious observations using default parameters with a false discovery rate (Benjamini-Hochberg FDR) correction significance threshold of 0.05. Note that ANCOM runs a bunch of pairwise tests. Each sub-hypothesis is structured as follows (for the 2-class case): $H_{0}\left( ij \right) :\mathrm{mean}\left\{ \log\left( x_{i}/x_{j} \right) \right\}=\mathrm{mean}\left\{ \log\left( y_{i}/y_{j} \right) \right\} ,$ where $x_{i}$ is taxon-$i$’s abundance in sample-$x$, $y_{i}$ is species-$i$’s abundance in sample-$y$, etc. Here, the W-score of a taxon just counts how many times $H_{0}\left( ij \right)$ is rejected for taxon-$i$. The higher the W-score, the more significantly differential is taxon-$i$.

**Random Forest classifier construction**

The random forest was also used to select differential species between two cohorts, and to verify the key discriminatory species which selected by random forest analysis^15, 16^. For microbiome data, each species was considered as a feature. For the selection of features, first, all the features were taken as training datasets with random forest algorithm using rfcv function in an R package ‘randomForest’ (V3.3.1) and then each feature’s importance score was calculated through permuting values of this feature and then calculating and normalizing the difference of out-of-bag errors before and after a permutation. Features were added one by one according to importance score of the feature (with descending order). The data is split into a training set and a test set, with 70% of the data forming the training data and the remaining 30% forming the test set. Ten times cross validation for 500 times was performed to sift through the minimum species combination with the lowest error rate and the lowest number that the mean prediction accuracy reaching the optimal value. Receiver operating characteristics (ROC) analysis was then performed to measure the quality of the classification models by the R software package pROC (V1.16.2)^17^. ROC curves results were plotted manually by the true positive rate against the false positive rate. ROC curves were constructed, and the area under the curve (AUC) was used to designate the ROC effect.

**Microbial correlation network analysis**

To further assess the potential interactions among microbial community members, network analysis was conducted. The correlation networks were constructed using SparCC (sparse correlations for compositional data, V1.1.0, https://github.com/luispedro/sparcc). Significant interactions were determined by the bootstrapped results (n = 100) using the script PseudoPvals in SparCC ^18^. The false discovery rate (Benjamini-Hochberg FDR) method was used to adjust the *P* values for multiple test correction. Significant correlations with absolute sparse correlations |r| > 0.3 and adjusted *P* < 0.05 were visualized using Gephi (https://gephi.org/). The larger size of nodes represents the more relative abundance species. Highly connected species taxa in each module can be considered as keystone taxa due to their central position in a microbiota network.

**Reference**

1. Ma JE, Jiang HY, Li LM, Zhang XJ, Li GY, Li HM, et al. The Fecal Metagenomics of Malayan Pangolins Identifies an Extensive Adaptation to Myrmecophagy. Front Microbiol 2018; 9:2793.

2. Li R, Yu C, Li Y, Lam TW, Yiu SM, Kristiansen K, et al. SOAP2: an improved ultrafast tool for short read alignment. Bioinformatics 2009; 25:1966-7.

3. Law J, Jovel J, Patterson J, Ford G, O'Keefe S, Wang W, et al. Identification of hepatotropic viruses from plasma using deep sequencing: a next generation diagnostic tool. PLoS One 2013; 8:e60595.

4. Qin N, Yang F, Li A, Prifti E, Chen Y, Shao L, et al. Alterations of the human gut microbiome in liver cirrhosis. Nature 2014; 513:59-64.

5. Wang W, Zheng S, Li L, Yang Y, Liu Y, Wang A, et al. Comparative metagenomics of the gut microbiota in wild greylag geese (Anser anser) and ruddy shelducks (Tadorna ferruginea). Microbiologyopen 2019; 8:e00725.

6. Zhu W, Lomsadze A, Borodovsky M. Ab initio gene identification in metagenomic sequences. Nucleic Acids Res 2010; 38:e132.

7. Fu L, Niu B, Zhu Z, Wu S, Li W. CD-HIT: accelerated for clustering the next-generation sequencing data. Bioinformatics 2012; 28:3150-2.

8. Langmead B, Trapnell C, Pop M, Salzberg SL. Ultrafast and memory-efficient alignment of short DNA sequences to the human genome. Genome Biol 2009; 10:R25.

9. Urban M, Cuzick A, Seager J, Wood V, Rutherford K, Venkatesh SY, et al. PHI-base: the pathogen-host interactions database. Nucleic Acids Res 2020; 48:D613-D20.

10. Huson DH, Auch AF, Qi J, Schuster SC. MEGAN analysis of metagenomic data. Genome Res 2007; 17:377-86.

11. Francis OE, Bendall M, Manimaran S, Hong C, Clement NL, Castro-Nallar E, et al. Pathoscope: species identification and strain attribution with unassembled sequencing data. Genome Res 2013; 23:1721-9.

12. Schloss PD, Westcott SL, Ryabin T, Hall JR, Hartmann M, Hollister EB, et al. Introducing mothur: open-source, platform-independent, community-supported software for describing and comparing microbial communities. Appl Environ Microbiol 2009; 75:7537-41.

13. Segata N, Izard J, Waldron L, Gevers D, Miropolsky L, Garrett WS, et al. Metagenomic biomarker discovery and explanation. Genome Biol 2011; 12:R60.

14. Mandal S, Van Treuren W, White RA, Eggesbo M, Knight R, Peddada SD. Analysis of composition of microbiomes: a novel method for studying microbial composition. Microb Ecol Health Dis 2015; 26:27663.

15. Breiman L. Random forests. Machine learning 2001; 45:5-32.

16. Cao Y, Wang L, Ke S, Villafuerte Galvez JA, Pollock NR, Barrett C, et al. Fecal Mycobiota Combined With Host Immune Factors Distinguish Clostridioides difficile Infection From Asymptomatic Carriage. Gastroenterology 2021; 160:2328-39 e6.

17. Robin X, Turck N, Hainard A, Tiberti N, Lisacek F, Sanchez JC, et al. pROC: an open-source package for R and S+ to analyze and compare ROC curves. BMC Bioinformatics 2011; 12:77.

18. Friedman J, Alm EJ. Inferring correlation networks from genomic survey data. PLoS Comput Biol 2012; 8:e1002687.
